# An examination of cancer epidemiology studies among populations living close to toxic waste sites

**DOI:** 10.1186/1476-069X-7-32

**Published:** 2008-06-26

**Authors:** Mark B Russi, Jonathan B Borak, Mark R Cullen

**Affiliations:** 1 Occupational and Environmental Medicine Program, Yale University School of Medicine, 135 College Street, New Haven, CT 06510, USA

## Abstract

**Background:**

Toxic waste sites contain a broad range of suspected or confirmed human carcinogens, and remain a source of concern to many people, particularly those living in the vicinity of a site. Despite years of study, a consensus has not emerged regarding the cancer risk associated with such sites.

**Methods:**

We examined the published, peer-reviewed literature addressing cancer incidence or mortality in the vicinity of toxic waste sites between 1980 and 2006, and catalogued the methods employed by such studies.

**Results:**

Nineteen studies are described with respect to eight methodological criteria. Most were ecological, with minimal utilization of hydrogeological or air pathway modeling. Many did not catalogue whether a potable water supply was contaminated, and very few included contaminant measurements at waste sites or in subjects' homes. Most studies did not appear to be responses to a recognized cancer mortality cluster. Studies were highly variable with respect to handling of competing risk factors and multiple comparisons.

**Conclusion:**

We conclude that studies to date have generated hypotheses, but have been of limited utility in determining whether populations living near toxic waste sites are at increased cancer risk.

## Background

Environmental epidemiological studies of communities surrounding toxic waste sites suffer from a range of limitations, some of which may predispose to missing an important effect, while others may predispose to the finding of spurious results. For example, due to the relative rarity of most cancers, large numbers of individuals must be exposed to a carcinogen for a long period of time for its effect to be perceived in an epidemiological study, and studies carried out in single communities over a limited number of years may lack the power necessary to detect such effects. On the other hand, studies of single communities in the vicinity of a toxic waste site may be undertaken after a cancer excess has been observed, predisposing to the publishing of studies from communities where excess cancers were already known to exist.

Because tens of millions of Americans live within a few miles of a toxic waste site, cancer excesses would be expected to occur with some frequency in such communities due to the same random fluctuations which produce cancer clusters in communities which do not contain toxic waste sites. Caution would need to be exercised in drawing conclusions based on the single studied community if for every study undertaken to follow up an excess rate of cancer in a community with a hazardous waste site, there are many more communities with similar sets of exposures which were not studied because a cancer cluster did not appear.

Other limitations derive from the challenges of exposure assessment, competing risk factor characterization, and multiple comparisons. An ideal study would assess accurately exposure levels at waste sites over long periods of time, and model exposure pathways from the waste site to the target population, or measure individual exposure levels. Because most residential exposures to toxic wastes occur at very low levels, the accurate quantification of competing cancer risk factors at the individual level, such as smoking, occupation, socioeconomic status, alcohol use, family/ethnic history, diet, etc, would also be of importance to avoid confounding. Finally, because most cancer incidence or mortality studies examine multiple tumors in multiple age groups, a study generating hundreds of comparisons requires some strategy to adjust for the fact that a certain number of positive associations would be expected to occur due to chance alone.

The methodological requirements outlined above would be challenging under most circumstances. Detailed measurements of toxic chemicals at waste sites over long periods of time are rarely available, complex hydro-geologic or air pathway modeling is resource intensive and challenging, and individually measured exposure levels are nearly impossible to obtain for community residents over lengthy periods of exposure. Detailed, individually ascertained data regarding competing risk factors also do not generally exist for large residential populations.

## Methods

We carried out Medline searches of the peer-reviewed English language medical literature covering the period from January, 1980, to June, 2006, using the keywords "toxic waste sites" and "cancer". Articles were also identified from published reviews. Selection criteria for inclusion were: 1) the study addressed either cancer incidence or cancer mortality as an endpoint, 2) the study was carried out in a community or a set of communities containing a known hazardous waste site, 3) the study had to address exposure from a specific waste site, rather than exposure from a contaminated water supply which may have resulted from multiple point sources. It is worthy of note that we did not include the well publicized and important groundwater contamination which occurred in Woburn, Massachusetts (where contaminants seeped into the groundwater supply from several industrial sources), due to this latter criterion. A total of 19 studies published from 1980 to 2006 met the inclusion criteria.

Several parameters were set up to assess study quality. The first was whether the study employed an ecological exposure assessment, or whether modeling or measurement of individual exposures was carried out. Examples of ecological exposure assessments would include assignment of exposure based on residence in a community, or residence in an area defined by proximity to a waste site. Examples of individual exposure assessments would include measurements at the residential site of tap water, soil, airborne contamination levels, or biomonitoring data combined with information on patterns of tapwater use, play patterns among children, or residence time at a specific address. For tapwater exposure, approximations of individual exposure levels could have been generated by detailed hydrogeological modeling based on data from a large number of test sites within the water distribution system, combined with information regarding individual tap water consumption. For soil exposure, approximations of individual exposure levels could have been generated by an assessment of individuals' play patterns combined with information regarding chemical contamination levels at neighborhood playgrounds, ball fields, or other gathering places for youth. For airborne exposure, approximations of individual exposure levels could have been generated by an assessment of the number of hours spent by an individual in specific areas, combined with measurement of airborne contaminants in those areas.

The second assessment criterion was whether or not the study was undertaken in response to a perceived cancer cluster in the community. This criterion was established in response to the concern that a certain number of cancer clusters occur by chance alone, and that communities experiencing such clusters may be more likely to undergo study than communities in which clusters have not been identified. Studies were not considered to be post-cluster analyses if the identification of an excess of health effects other than cancer provided the impetus for a study to be undertaken.

The next criterion addressed the amount of information provided regarding specific chemical contaminants at the waste site. Although waste sites may contain hundreds of chemicals, emphasis was placed on whether one or more probable or established carcinogens had been measured. Chemicals of particular concern included chromium, arsenic, lead, cadmium, nickel, benzene, perchlorethylene, trichloroethylene, methylene chloride, vinyl chloride, chloroform, ethylene dichloride, and polychlorinated biphenyls. Our assessment included whether the investigators measured soil or water levels at the waste site, merely enumerated chemicals, or did not provide specific information regarding the chemicals at the site.

Because the greatest potential for human exposure to chemicals emanating from a waste site could come from consumption of or aerosol exposure to contaminated tapwater, we reviewed studies to determine whether a potable water supply was contaminated by the waste site. It was expected that studies of multiple sites might include some sites with water contamination and others with no known contamination.

The next criterion addressed whether the study attempted to model exposure to waste site toxins through some method more refined than the assignment of exposure based on residence in the community or geographical area in which a waste site was located. Modeling could have included hydrogeologic assessments of contaminant delivery through tapwater, assessments of airborne contaminant spread based on air sampling and wind pattern data, estimations of soil contamination based on soil sample data from representative sites within the affected area.

A criterion also was established to assess whether risk factors other than proximity to a waste site were addressed. Such competing risk factors could have included smoking, occupational exposures, alcohol consumption, and demographic characteristics such as age, gender, ethnicity, urban residency, household size, income, and education. A priori concern was great regarding socioeconomic markers, since those living in the immediate vicinity of a toxic waste site may be of lower socioeconomic status than those living in surrounding areas [3]. If competing risk factors were addressed, it was noted whether such risk factors were evaluated based on individual data, or aggregated community-level information.

Because the most precise classification of individual contaminant exposure would require measurement of contaminants at the site of exposure, we screened studies to determine whether any had assessed contaminant levels in the home. Measurements could have included tap water contaminant levels, backyard soil measurements, or indoor air contaminant measures.

Finally, because many investigators undertook analyses of many subpopulations for a range of distinct cancers, studies were assessed to determine whether statistical adjustments for multiple comparisons were made.

### Study Summaries

Summaries and brief commentary regarding the studies meeting inclusion criteria appear in the attached appendix (see additional file 1).

## Results and Discussion

Results are summarized in the table (see additional file 2).

Sixteen of the nineteen reviewed studies were ecological surveys, in which cancer incidence or mortality in regions defined as exposed or unexposed was assessed without attention to individual variations of exposure among the afflicted and non-afflicted. Other study methodologies included a case-control study in Niagara County, New York; a cross-sectional survey in a rural New Jersey community; and an analysis of migration patterns among children in Great Britain [10,18,20].

Because exposure levels may have varied considerably among residents in areas containing toxic waste sites, (and therefore such community residents would represent a substantially heterogeneous group) the capacity of the reviewed studies to demonstrate cancer risk from low-level environmental exposures to waste site chemicals was limited. At the same time, it is scarcely realistic to expect individual-level analyses of actual exposure to have been carried out over long latency periods. There have been no detailed environmental data gathered on which to base such assessments, and the myriad of individual factors which modify exposure to environmental chemicals, such as tapwater consumption habits, time spent indoors and outdoors, housing characteristics, and work habits, adds layers of complexity. The lack of individual-level analysis of exposure to waste site chemicals is also problematic because any increase in cancer risk from waste site chemicals would be expected to be small in comparison to effects from other cancer risk factors, such as smoking. Without data assessing whether individuals with cancer were more highly exposed to waste site chemicals than were individuals without cancer, the conclusions which can be inferred are limited.

Elements of an individual-level assessment of exposure to waste site toxins might have included measurement of toxins at the waste site, air or tap water measurement in the home of waste site chemicals, biomonitoring data, measurement of chemicals in a potable water supply combined with detailed information regarding distribution of the supply, as well as detailed individual data on daily activity patterns, water consumption, etc. Of the reviewed studies, five contained information regarding specific contaminants at the waste site, with a small number of measurements reported [5,6,11,18,21]. One study measured contaminants in exposed homes [21]. Concentrations were found to be similar to homes defined as unexposed.

A contaminated water supply may serve as an important conduit for exposure of local populations to waste site chemicals, through both drinking water consumption and aerosolization of chemicals while showering or cooking [1,12,13]. Most studies did not address whether such contamination had occurred; one study measured water contaminants [18]. For several studies, the potable water supply was not contaminated or residents received water from other sources.

None of the reviewed studies modeled exposures based on a combination of representative measurements and hydrogeologic predictions, patterns of water consumption, estimates of time spent indoors vs. outside, exposure to soil, or other indices of interpersonal variation in exposure. Nearly all studies defined residents as exposed or non-exposed based on residence in a region containing a waste site. A few assigned low, medium or high exposures based on distance from a site. One study incorporated hydrogeologic monitoring, requiring a gradient of groundwater flow from the dumpsite to the residential area [18].

Assessment of competing cancer risk factors was considered an important element in the evaluation of studies, particularly given the likelihood that populations in the immediate vicinity of a toxic waste site may be of lower socioeconomic status than those more distant from such sites. Our review revealed that approximately half the studies assessed competing cancer risk factors. For several studies, risk factors such as per capita income, rate of adverse pregnancy outcomes, concentrations of chemical industries, population density, and urbanization indices were characterized at the regional level. For potentially important competing risk factors such as smoking and alcohol consumption, reliable data generally do not exist to characterize exposure at a regional level, and substantial individual variation in exposure limits the value of an aggregate exposure index. Four studies appear to have gathered data at the individual level regarding other potential cancer risk factors [2,6,18,20]. Those data included age, family income, smoking and alcohol consumption, ethnicity, education level, occupational exposure, and diet.

For most of the reviewed studies, there did not appear to have been a cancer cluster recognized prior to the study's being undertaken. For some studies, local concern had existed regarding health outcomes associated with a toxic waste site, but no cancer cluster had been formally recognized at the time study was undertaken [2,23]. For others, elevated cancer rates in the study area were recognized before the studies began [11,14,20]. For one group of studies, a death certificate analysis had revealed regionally elevated cancer mortality during years prior to the study years [15-17,19].

Because several studies undertook assessments of many different cancers and included a range of sub-group analyses, we assessed for each whether multiple comparisons were made, and whether investigators incorporated a means to adjust for the probability that some associations would occur by chance alone. Studies differed considerably along this parameter. Several studies employed p-values of less than 0.05 to compensate for multiple comparisons. Examples included a study of the Drake Superfund site, which included more than 700 comparisons, employing a p-value of 0.025 [4]; and a study of gastrointestinal cancer mortality with more than 400 comparisons, employing a p-value of 0.01 [19]. One study employed a Bonferroni adjustment. Several studies did not appear to have included a statistical adjustment for multiple comparisons [5,6,8,11,21-23].

## Conclusion

The study of whether an individual's risk of cancer is heightened by environmental exposure to chemicals emanating from toxic waste sites is extremely challenging. To date, epidemiological studies of populations living in the vicinity of a toxic waste site have not produced evidence of a quality that most epidemiologists would consider adequate to establish a causal link between toxic waste exposures and cancer risk. Studies have not included individual assessments of chemical exposure from toxic waste sites, few included measurements at waste sites, and only one included measurements of chemicals in the home. Exposure models have generally been limited to a judgment of whether individuals were exposed or not exposed based on residential proximity to a waste site. Few studies include individual data regarding competing risk factors, many do not address whether a potable water supply was contaminated by a waste site, and several include a large number of comparisons without statistical adjustment.

If there is a cancer risk to populations living in the vicinity of toxic waste sites, it is likely to be of a magnitude not detectable via methodologies utilized to date. Without individual-level data on specific chemical exposures and competing risk factors over long latency periods, the few associations seen to date are more likely due to multiple comparisons and presence of competing risk factors than to the unmasking of a true exposure effect. Lack of a consistently occurring risk for some specific tumor across multiple studies further suggests this. If progress is to be made in determining whether environmental exposure to toxic waste site chemicals increases cancer risk, it awaits the assembly of a massive collection of individual-level exposure data to toxic waste site chemicals over long periods of time and a detailed individual-level assessment of competing cancer risk factors. The likelihood is low that so detailed an assessment will ever be undertaken.

While epidemiological studies may not have produced convincing evidence for a causal association between living in the vicinity of a toxic waste and specific cancer outcomes, we believe that data from representative populations in the vicinity of toxic sites should continue to be gathered and monitored for the same reasons that cancer outcomes are more generally monitored in tumor registries, e.g. to estimate rates at particular periods of time for subsequent comparison, to furnish ongoing disease surveillance of possibly exposed (and concerned) residents, or to generate hypotheses for future research. The discovery of a consistently elevated incidence of a cancer across multiple exposed populations will never occur unless monitoring of such populations is ongoing. Such a discovery would imply a larger effect, one potentially perceptible despite inherent limitations of the data and methodologies used to date to study populations living in the vicinity of toxic waste sites.

## Competing interests

The literature review and data analysis underlying this study was supported in part by the City of New York.

## Authors' contributions

MR reviewed the primary literature, participated in the development of evaluation criteria, and drafted the manuscript, JB reviewed the primary literature, participated in the development of evaluation criteria, and contributed to the drafting of the manuscript, MC reviewed the primary literature and contributed to the drafting of the manuscript.

## Supplementary Material

Additional File 1 Appendix. Summaries and brief commentary regarding studies meeting inclusion criteria. Click here for file

Additional File 2 Table. Criteria applied to individual studies Click here for file
