# Supplementary material for: An examination of cancer epidemiology studies among populations living close to toxic waste sites
Source: Environ Health. 2008 Jun 26;7:32. doi: 10.1186/1476-069X-7-32 (PMC2443363; doi:10.1186/1476-069X-7-32)
Supplement: Additional File 1 — Appendix. Summaries and brief commentary regarding studies meeting inclusion criteria. [file 1476-069X-7-32-S1.doc]

**Appendix**

Janerich DT, Burnett WS, Feck G, Hoff M, Nasca P, Polednak AP, Greenwald P, Vianna N. Cancer incidence in the Love Canal area. Science 1981;212(4501):1404-1407.

In this paper, the authors summarized cancer incidence in the census tract surrounding the Love Canal for the period 1955-1977. Standardized incidence ratios were calculated for each cancer site in the Love Canal tract, based on New York State (exclusive of New York City) cancer incidence rates and census data. In addition, New York State Cancer Registry data were used to compare cancer incidence for five different age groups at ten major cancer sites for each of the 25 census tracts in the city of Niagara Falls. For males and for females, fifty age/tumor site combinations for each census tract were rank ordered by incidence rate.

The study was undertaken in response to public concern about health hazards associated with buried wastes, but did not appear to have followed discovery of a cancer cluster. Exposure to the Love Canal waste site was based on residence in the same census tract and on having a street address within “close proximity” to the dump site. Details for the proximity analysis were not provided. The article did not report measurements of chemicals at the waste site, in residences, or in other locations. The authors did not comment regarding whether residents were exposed to a contaminated potable water supply. No assessment of competing cancer risk factors was carried out. An estimated 2500 comparisons were carried out in the study (gender-specific cancer incidence rates for 10 major cancer sites and 5 age groups for each of 25 census tracts). There did not appear to have been a statistical adjustment for multiple comparisons.

## Results

Rates for the Love Canal census tract were in the highest quintile 9 out of 50 times for males, and 8 out of 50 times for females. There was no increase in lymphoma or leukemia in the Love Canal tract. The SIR for female liver cancer only during the period 1955-1965 was increased (p<0.05), but the residences of these patients were reportedly not close to the dumpsite. The only other tumor for which rates were statistically significantly elevated in the Love Canal tract was respiratory cancer. However, the Love Canal tract and seven other census tracts as well as the city of Niagara Falls in total had statistically significantly higher respiratory cancer rates among males. Respiratory cancer SIRs were significantly elevated in one other Niagara Falls census tract for females. Street addresses of the patients with respiratory cancer indicated that there was no tendency for cases to be located in closer proximity to the dump site than non-cases.

There was a generally increased incidence of respiratory cancer among males in Niagara Falls, but there did not appear to be higher rates among those living in close proximity to Love Canal.

**Najem GR, Thind IS, Lavenhar MA, Louria DB. Gastrointestinal cancer mortality in New Jersey counties, and the relationship with environmental variables. Int J Epidemiol 1983;12:276-289.**

In this ecological survey, the authors examined gastrointestinal malignancy in New Jersey. Esophageal, gastric, pancreatic, colonic and rectal cancers over the period from 1968 to 1977 in 21 New Jersey counties were compared to national rates and to New Jersey rates from 1950-1969. The correlation between GI cancer mortality rates and the following variables, assessed at the aggregate level, was also studied: presence of toxic chemical waste disposal sites, birth defects rates, low birth weight rates, infant mortality rates, chemical industry concentrations, percentage of population employed in the chemical industries, population density, urbanization indices, and annual per capita income.

The study appears to have been undertaken in response to unusually high cancer mortality rates in New Jersey during the period from 1950-1969. The index of exposure to toxic waste sites was based on the number of such sites per 100 square miles of land surface area in each county. No analysis of residential proximity or modeling of waste site exposures was undertaken. Chemical measurements were not carried out, nor was there information regarding contamination of potable water.

No individual assessment of competing risk factors was carried out, though average per capita income, low birthweight rate, infant mortality rate, birth defect rate, chemical industry concentration, percentage of population employed in chemical industry, population density, and urbanization indices were calculated for each county.

Since five cancer sites were analyzed within 4 population subgroups living in 21 different counties, a total of 420 comparisons were made in the study. A p-value less than 0.01 determined statistical significance.

## Results

Age-adjusted GI cancer mortality rates (all sites combined) were higher than national rates in 20 of 21 counties. The associations most frequently found were with urbanization indices, population density and to a lesser extent with toxic waste dump sites. Few significant associations were found with low birth weight, infant mortality, birth defects or with percentage of persons employed in chemical industries. No associations with presence of toxic waste dump sites were seen for pancreatic cancer or rectal cancer. An association was seen for white males only with colon cancer, for white females and non-white females for stomach cancer, and for white males and females and non-white males for esophageal cancer.

Cancer rates in 20 of 21 New Jersey counties exceeded national rates. Population density and degree of urbanization were frequently associated with higher cancer mortality rates in specific subgroups. An association between the presence of toxic waste dumps in the county and cancer mortality in certain subgroups occurred less consistently. No individual level data were gathered regarding the risk factors examined or other important exposures such as alcohol/tobacco consumption. The authors commented that alcohol and tobacco consumption needed to be assessed as possible risk factors for elevated cancer mortality in the counties, and others have shown correlations between urbanization and tobacco/alcohol consumption.

Najem GR, Louria DB, Najem AZ. Bladder cancer mortality in New Jersey counties, and relationship with selected environmental variables. Int J Epidem 1984;13:273-282.

In another ecological survey, the authors examined via similar analytical methods bladder cancer mortality in New Jersey. Bladder cancers over the period from 1968 to 1977 in 21 New Jersey counties were compared to national rates and to New Jersey rates from 1950-1969. The relationship between bladder cancer mortality rates and the following variables, assessed at the aggregate level, was studied: birth defects rates, low birth weight rates, infant mortality rates, percentage of population employed in the chemical industries, population density, urbanization indices, chemical toxic waste disposal sites and annual per capita income.

The study appears to have been undertaken in response to death certificate studies from 1950-1969 revealing New Jersey to have the highest overall cancer and bladder cancer mortality rate. The index of exposure to toxic waste sites was based on the number of such sites per 100 square miles of land surface area in each county. No analysis of residential proximity was undertaken, nor was there measurement of waste site toxics. No information is provided regarding whether potable water was contaminated.

Individual assessment of competing risk factors was not carried out, though average per capita income, low birthweight rate, infant mortality rate, birth defect rate, chemical industry concentration, percentage of population employed in chemical industry, population density, and urbanization indices were calculated for each county.

A total of 84 comparisons were carried out (four population subgroups in 21counties). A range of p-values was employed.

## Results

Age-adjusted bladder cancer mortality rates were higher than national rates in 20 of 21 counties. A statistically significant correlation between bladder and lung cancer mortality in all 21 New Jersey counties was found among women, but not among men, suggesting exposure among women to a common carcinogen such as tobacco. A statistically significant association was found between bladder cancer mortality in individual counties and the percentage of the adult population working in the chemical industry, suggesting at the aggregate level a role of occupational exposure. There was no statistically significant association between bladder cancer mortality and urbanization index, population density, annual per capita income or concentration of toxic waste disposal sites within the 21 New Jersey counties.

**Budnick LD, Sokal DC, Falk H, Logue JN, Fox JM. Cancer and birth defects near the Drake superfund site, Pennsylvania. Arch Env Hlth 1984;39:409-413.**

This paper described an ecological survey of cancer mortality and birth defects in Clinton County, which contains the Drake Superfund site. Cancer mortality and birth defects in Centre, Lycoming and Union Counties (located within 20 miles of the Drake Superfund site), in Pennsylvania, and in the United States were used as comparison populations. The Drake Superfund site was contaminated with beta-naphthylamine, benzidene, and benzene. Cancer mortality rates for the decades of the 1950s, 1960s, and 1970s were reviewed. The incidence of specific birth defects for the period 1973-1978 in Clinton County alone was also tabulated.

Exposure to the dump site was based on residence in Clinton County. There does not appear to be an analysis of residential proximity to the waste site, nor any modeling of exposure. The authors did not report measurements of chemicals, or whether potable water was contaminated. Competing risk factors were not assessed. Thirty cancer sites were specified for males and 31 for females. Since cancer mortality rates were calculated for each of the three decades and for each of the four counties, a total of 360 rates for specific primary tumors among men, and 372 rates for women would have been compiled. A p-value of 0.025 was employed for significance testing.

## Results

Among the 732 male and female rates, 33 (or approximately 4.5% of the total number of rates) were statistically significantly elevated compared to national, age-adjusted, sex-, race- and site-specific cancer mortality rates.

Among the significantly elevated rates was bladder cancer mortality among men during the decade of the 1970s in Clinton County. Bladder cancer was of initial interest due to its association with some of the substances identified at the Drake site (beta-naphthylamine and benzidine). There was no corresponding elevation among women, nor were bladder cancer rates among men in Clinton County elevated during the decades of the 1950s or 1960s. Bladder cancer was not elevated among men in any of the other three counties surrounding the Drake site. The authors pointed out that the pattern of disease occurrence did not suggest a general environmental exposure, but that occupational exposures may have contributed to increased bladder cancer rates among men in Clinton County during the 1970s.

**Najem GR, Louria DB, Lavenhar MA, Feuerman M. Clusters of cancer mortality in New Jersey municipalities; with special reference to chemical toxic waste disposal sites and per capita income. Int J Epid 1985;14:528-537.**

In this study, Najem et al surveyed mortality due to 13 specific cancers during the period 1968-1977 in 194 New Jersey municipalities with respect to the distribution of chemical toxic waste disposal sites, annual per capita income, birth defects, low birth weight and infant mortality. Rates were compared to the age-, sex-, and race-adjusted U.S. population. Cancers studied were esophagus, stomach, pancreas, colon, rectum, larynx, lung, bladder, prostate, cervix, uterus, ovary and female breast.

The study appears to have been undertaken in response to death certificate studies from 1950-1969 revealing New Jersey to have the highest overall cancer and bladder cancer mortality rate. The index of exposure to toxic waste sites was based on the number of such sites per 100 square miles of land surface area for New Jersey municipalities with populations over 10,000. No analysis of residential proximity was undertaken, nor was there measurement of waste site toxics. Information is not provided regarding whether potable water was contaminated.

Individual assessment of competing risk factors was not carried out, though average per capita income, low birthweight, infant mortality, and birth defect rates were calculated for each municipality.

A total of 7760 comparisons were made. A p-value of 0.0005 was used for significance testing.

## Results

In 51 (24%) of the 194 municipalities with populations of 10,000 or more people, in 16 of 21 New Jersey counties, 146 age-adjusted death rates among white males and females exceeded the national rates by at least 50% and the observed number of deaths were highly significantly (p<0.0005 or smaller) greater than the expected deaths for at least one cancer. Twenty-three municipalities in 10 New Jersey counties contained sufficient excesses of cancers to fulfill the authors’ definition of clusters. Of the 98 clusters located in those 23 municipalities, 72% involved the gastrointestinal tract, while the remaining 28% involved the larynx, bladder, uterus, ovary and breast. Of the 23 municipalities, 16 were located in the heavily industrialized northeast corridor of the State. Those same 16 municipalities contained 18% of the total New Jersey population.

A correlation analysis between cancer mortality and a number of variables revealed a significant positive association with the presence of a chemical toxic waste dump site for one or more subgroups in 8 of the 12 cancers studied. Significant correlations were also found between cancer mortality and low birth weight, birth defects, infant mortality, and annual per capita income.

For presence of chemical toxic waste dumpsites, correlation coefficients ranged from 0.14 to 0.29. Of note, except for 3 of the 20 significant negative correlations with income, all of the variables studied accounted for less than 10% of the variance, suggesting that a relatively small proportion of the differences among cancer mortality rates could be attributed to presence of toxic chemical waste dump sites, per capita income, higher rates of low birth weight, birth defects, or infant mortality in the municipality.

**Najem GR, Greer TW. Female reproductive organs and breast cancer mortality in New Jersey counties and the relationship with certain environmental variables. Preventive Medicine 1985;14:620-635.**

This was an ecological survey of cancers of the cervix, uterus, ovary and breast among New Jersey women over the period 1968-1977, compared to age-specific U.S. rates.

The study appears to have been undertaken in response to death certificate studies from 1950-1969 revealing New Jersey to have the highest overall cancer mortality rate and to have very high mortality rates from cancers of the female reproductive organs and breast. The index of exposure to toxic waste sites was based on the number of such sites per 100 square miles of land surface area in each county. No analysis of residential proximity was undertaken, nor was there measurement of waste site toxics. Information is not provided regarding whether potable water was contaminated.

Individual assessment of competing risk factors was not carried out, though average per capita income, low birthweight rate, infant mortality rate, birth defect rate, chemical industry concentration, percentage of population employed in chemical industry, population density, urbanization indices were calculated for each county.

A total of 168 comparisons were made. A p-value of 0.05 was used for significance testing, but the authors reported that greater credence was given to associations with a p-value less than 0.0005.

## Results

The authors reported that for 19 of 21 New Jersey counties, cancer mortality rates for all four sites combined exceeded national rates. Statistically significant positive correlations were found between breast cancer mortality and annual per capita income, urbanization index, chemical toxic waste disposal sites, and population density among whites. No correlations were found between the other cancers and presence of chemical toxic waste disposal sites.

**Baker DB, Greenland S, Mendlein J, Harmon P. A health study of two communities near the Stringfellow waste disposal site. Archives of environmental health 1988;43:325-334.**

In this study, a health survey questionnaire was administered to 2039 people living in 606 households near the Stringfellow hazardous waste site in Riverside County, California, or in a non-exposed reference community. Data consisted of questionnaire results, medical records, and birth and death certificates. Residents were considered to have a high likelihood of exposure, a low potential for exposure, or no potential exposure, based on residence in one of three communities. Measures of residential proximity to the waste site were not undertaken, nor was exposure modeling. A potable water supply was contaminated, but toxics were not specifically measured. The competing risk factors of age, gender, ethnicity, religion, household size, household income, respondent education, occupation, and smoking were assessed individually through the questionnaire. Only two cancer mortality comparisons were carried out (overall cancer mortality in the highly exposed area vs. non-exposed, overall cancer mortality in low exposure area vs. non-exposed)

## Results

Subjective outcomes, such as self-reported diseases and symptoms, were reported more frequently among residents living closer to the waste site, while objective outcomes, such as mortality, cancer incidence and pregnancy outcomes, were not shown to differ according to waste site proximity.

There were no statistically significant excesses of overall mortality, cancer mortality, cardiovascular mortality, or cancer incidence in the exposed population compared to the control population. In contrast, rate ratios were greater than 1.5 for 5 of 19 self-reported diseases on the questionnaire (ear infections, bronchitis, asthma, angina pectoris, and skin rashes) among study subjects living in areas close to the waste site. Similarly, prevalence odds ratios for 23 symptoms were uniformly greater than 1.0 among all subjects living close to the waste site, and 8 symptoms had odds ratios greater than 1.5 (blurred vision, pain in ears, daily cough for more than a month, nausea, frequent diarrhea, unsteady when walking, and frequent urination).

The authors suggested that those living near the site may have had increased recall or perception of health conditions. To test this, respondents were separated into those who perceived a great health threat from proximity to the waste site vs. those who didn’t. There was substantial attenuation of symptom prevalence odds ratios when analysis was restricted to subjects who did not have the “highest perception of threat” from being close to the waste site. The authors also commented that there was no toxicological mechanism to explain a uniform elevation of odds ratios for every one of 23 symptoms among subjects living in close proximity to the waste site.

**Griffith J, Duncan RC, Riggan WB, Pellom AC. Cancer mortality in U.S. counties with Hazardous waste sites and ground water pollution. Archives of Environmental Health 1989;44:69-74.**

The goal of this study was to determine whether excess cancer rates occurred at an increased frequency in U.S. counties with hazardous waste sites contaminating a ground water supply. The authors identified 593 waste sites in 339 U.S. counties in 49 states with evidence of contaminated ground drinking water providing a sole source water supply. For each identified county, age-adjusted cancer mortality rates among white men and women for 13 different cancers during the period from 1970-1979 were extracted from the EPA/NCI U.S. Cancer and Mortality Trends database. Each U.S. county was coded for each cancer as to whether there was “excess” mortality. (Definition of excess mortality is not provided in the paper.) The relative frequency of excess cancer mortality was compared between exposed (contained a hazardous waste site contaminating a public water supply) and non-exposed counties. Counties containing hazardous waste sites were not matched by degree of urbanization to counties not containing hazardous waste sites.

## The study addressed solely hazardous waste sites which contaminated a ground water drinking supply, and did not consider waste sites for which there was no evidence of ground water contamination, or sites where local residents derived their drinking water from other sources. Exposure to the waste site was assigned based on residence in a county containing a waste site. No proximity analysis or exposure modeling was undertaken. Testing was carried out to identify contaminated water supplies, but information regarding specific toxics or levels was not provided. No analysis of competing risk factors was carried out. Bonferroni adjustments were made for multiple comparisons.

## Results

The authors reported significant associations (p<0.002) between excess deaths and counties containing hazardous waste sites for cancers of the lung, bladder, esophagus, stomach, large intestine, and rectum for white males; and for cancers of the lung, breast, bladder, stomach, large intestine and rectum for white females, compared to all counties without hazardous waste sites.

The authors pointed out that individuals in hazardous waste site counties may have been exposed to chemical pollutants while working for companies that created the waste, through contamination of local food supplies, emissions into the ambient environment, and contaminated water. They also conjectured that hazardous waste sites may be located in areas that are more industrialized, less desirable for residence, or differ from other U.S. counties in other ways, leading to sizeable differences in personal habits, ethnic, and other factors linked with cancer risks between residents of exposed and non-exposed U.S. counties. Finally, the authors noted that the populations using contaminated ground drinking water may be very small compared with total county populations, and levels of contamination may be very low.

**Polednak A, Janerich DT. Lung cancer in relation to residence in census tracts with toxic-waste disposal sites: A case-control study in Niagara County, New York. Environmental Research 1989;48:29-41.**

This case-control study examined risk of lung cancer among residents of nine census tracts containing toxic-waste disposal sites in Niagara County, New York. The study was carried out in response to the finding of increased respiratory cancer incidence in a number of census tracts within Niagara Falls, NY, including the census tract containing the Love Canal site.

Residence within census tracts known to contain waste sites contaminated with one or more lung carcinogens was used as an index of exposure. Dumpsites in Niagara County were selected (prior to data analyses) because known or suspected lung carcinogens had been identified in them. Carcinogens included chromium compounds, asbestos, arsenic, coal tar, nickel, ionizing radiation from uranium oxides and radium, and vinyl chloride. No data were gathered regarding contaminant levels or residential proximity to the waste sites, nor were data available on migration of contaminants in soil, water or air. The authors did not report whether a potable water supply was contaminated.

The study comprised two parts, a death certificate study and a questionnaire-based study. For the death certificate study, a total of 339 deceased lung cancer cases were compared to 676 Niagara County controls with adjacent death certificate numbers, who died of other causes. Controls were matched by sex, age at death, year of death and race.

The questionnaire-based study compared results of 209 questionnaires completed by case informants (63.4% of 309 questionnaires mailed to informants) to results from 417 control informants’ questionnaires. Questionnaires assessed smoking history, residential history, medical history of selected respiratory diseases, diet prior to illness and years of education.

The study did not contain multiple comparisons.

## Results

No association between death from lung cancer and residence in one of the contaminated census tracts was found (odds ratio 0.95, 95% confidence interval: 0.65, 1.38).

The questionnaire study revealed that both history of ever smoking cigarettes and history of chronic lung disease were significantly more frequent in cases than controls. (Both at p < 0.001) Cases and controls did not differ significantly in history of ever having lived in selected census tracts with dumpsites of interest (23.4% among cases vs. 20.6% among controls; odds ratio 1.17, 95% CI: 0.78, 1.76) A logit-model analysis, which included age, residence in selected census tracts, and history of smoking 20+ cigarettes per day, showed smoking to be the only significant predictor of risk (odds ratio = 4.54).

**Muir KR, Hill JP, Parkes SE, Cameron AH, Mann JR. Landfill waste disposal: an environmental cause of childhood cancer? Paediatric and Perinatal Epidemiology 1990; 4:484-485.**

This brief report summarized a study of childhood cancer incidence in the vicinity of a toxic waste dump located in Great Britain. The study was undertaken by the West Midlands Regional Children’s Tumour Registry in response to concerns about childhood cancer among local residents. Childhood cancer incidence from 1980-1988 was examined in the population of the Ward containing the waste site, in the contiguous wards, and in a downwind region likely to have been affected by airborne dispersion of contaminants. Exposure was defined as residence in one of those three defined areas. Distances between specific residences and the waste site were not part of the analysis. The start date of 1980 was chosen to coincide with when regular dumping of waste began.

Measurements of specific toxics at the dump site or in homes were not undertaken. The authors did not report whether a potable water supply was contaminated. Competing risk factors were not assessed. Since childhood cancers as a group were analyzed, multiple comparisons were not carried out.

## Results

In the ward containing the dumpsite and in those wards contiguous to it, there were no statistically significant differences between observed and expected numbers of childhood cancers. The authors also reported that there were no unusual distributions of diagnoses. The study was limited by the relatively low numbers of expected and observed cases and a brief follow-up period.

**Mallin K. Investigation of a bladder cancer cluster in northwestern Illinois. American Journal of Epidemiology 1990;132 (Suppl 1):S96-S106.**

This was an investigation carried out in 8 counties of northwestern Illinois which experienced increased mortality rates from bladder cancer during the period from 1950 to 1979. The investigator carried out an incidence and mortality study of bladder cancer in the counties over the period from 1978 to 1985.

An index of exposure to a waste site was not incorporated into the study design. Standardized Mortality Ratios and Standardized Incidence Ratios were reported by county and zip code. No analysis of residential proximity to hazardous waste sites was carried out.

There was a contaminated public water supply, and measurements taken from the two contaminated wells revealed trichloroethylene levels in the range of 2 – 15 ppb (EPA drinking water standard 5 ppb). Other contaminants were within recommended limits, where applicable Illinois EPA primary drinking water standards existed. In-home measurements were not taken. Competing risk factors were not assessed.

Standardized incidence ratios were calculated for 8 counties and 97 separate zip codes. A statistical correction for multiple comparisons was not made.

## Results

There was no elevation of age-adjusted bladder cancer standardized incidence ratios for any of the 8 counties. Standardized incidence ratios were also calculated separately for the 97 zip codes of the counties, and 2 were found to be elevated in a statistically significant way. In one of the two zip codes, bladder cancer incidence was significantly increased among both men and women. The investigator reported that a well had been closed due to contamination in that zip code, and that two other wells, which had trace contamination with trichloroethylene, tetrachloroethylene and other solvents, were within a half-mile of landfill site containing municipal and industrial wastes.

**Najem GR, Strunck T, Feuerman M. Health effects of a superfund hazardous chemical waste disposal site. Am J Prev Med 1994;10:151-155.**

This was a cross-sectional study of 358 households (1454 people) in a rural New Jersey community containing a single Superfund hazardous chemical waste dump site. The dump site was located on 15.5 acres of land with approximately 60 residential properties located within a half-mile of the site, and 19,700 residents living within 3 miles of the site. No chemical industries were located in the community. The study did not appear to have been initiated in response to a disease cluster.

Subjects residing 5 years or more in households within 1 mile of the waste site were considered exposed. A gradient of groundwater flow had to exist from the waste site to the residence. Chemicals identified in water sampling from test wells included methyl methacrylate, bis(2-ethyhexyl)-phthalate, di-n-butyl phthalate, benzene, toluene, ethylbenzene, and trichloroethylene. Gross alpha radiation at nearly three times the Federal and State Primary Drinking Water Standard of 15 picocuries/liter was detected. Subsurface soil sampling was performed at 15 on-site shallow test pits, revealing methyl methacrylate, bis(2-ethylhexyl)phthalate, benzene, toluene, methylene chloride, ethylbenzene and lead. Air sampling collected downwind was negative with the exception of two samples which demonstrated detectable levels of methyl methacrylate (0.03 and 4.95 ppm).

In-person interviews were conducted to assess on an individual basis competing risk factors including demographic information, occupation, environmental exposure, and personal medical history. Since the study examined overall cancer risk, statistical adjustment for comparisons of multiple tumors was not needed.

## Results

The only significant associations were a significantly higher prevalence of respiratory diseases lasting more than one month and a higher prevalence of seizures among the exposed group compared with the reference population. However, when the data were stratified by water supply or consumption of homegrown vegetables or cigarette smoking, the significant differences between exposed and comparison groups disappeared. The differences in prevalence of cancer, liver illnesses, and skin diseases between the exposed and comparison groups were not significant.

The study’s ability to detect cancer excesses was substantially limited by its small sample size.

**Goldberg MS, Al-Homsi N, Riberdy H. Incidence of cancer among persons living near a municipal solid waste landfill site in Montreal, Quebec. Arch Env Health 1995;50:416-424.**

This study sought to determine whether residents living close to the Miron Quarry municipal solid waste landfill site in Montreal, Quebec, had higher cancer incidences than residents in surrounding areas. The site is the third largest municipal solid waste landfill site in North America, and approximately 100,000 people live within 2 kilometers. Because Montreal residents obtain potable water from sources other than groundwater at or near the site, the primary health concern of local residents was air contamination from chemicals. Since 1980 gases emanating from the site have been trapped by a pipe network and combusted, though the system apparently has operated at low efficiency. A wide range of volatile organic compounds has been measured within the pipe network.

The Public Health Department was commissioned by the city of Montreal to carry out a cancer risk assessment of the site in response to concerns of local residents.

Low, medium, and high exposure zones were established based on proximity to the dumpsite. The high-exposure zone comprised the postal code in which the quarry was located and the postal code areas that bordered it. The high exposure zone was further divided into two subzones to take into account prevailing winds. The medium exposure zone included all postal codes that bordered the high-exposure zone on the north, south, and east sides. The low-exposure zone included postal codes northeast of the medium and high zones. Individual distances of residences from the waste site were not part of the analysis.

Cancer incidence rates were compared with various unexposed reference populations within Montreal. The primary reference population was all persons living in nonexposed areas on the island of Montreal. Because that group was sociodemographically not entirely comparable with the exposed populations, smaller reference groups which were more similar to the exposed populations were selected from it and compared to the exposed populations. However, those smaller reference groups also differed in some ways from the exposed populations. The exposed were considerably less likely to have received a university education, more likely to have had less than a 9^th^ grade education, and more likely to be of Italian descent. There was no control for smoking.

Lacking direct measurements of gases emanating from the site, proximity was used as a proxy of exposure. No attempt was made to employ modeling based on air current data, though the high exposure zone was divided in two parts based on prevailing winds. Data were not gathered at the individual level for competing risk factors. A total of 148 comparisons were made. No statistical adjustment for multiple comparisons was undertaken.

## Results

Seventeen cancer sites were evaluated among men for a total of 68 analyses, (taking into account low, medium, and high exposures); while 20 cancer sites were evaluated in women with a total of 80 analyses. The authors reported significant or nearly significant positive associations in the high-exposure zones for two sites of cancer in women (stomach, cervix) and four sites in men (stomach, liver and intrahepatic bile ducts, trachea/bronchus/lung, prostate). Excesses of trachea/bronchus/lung cancer occurred in the low and medium exposure zones, as well as in the high exposure zone. With 148 comparisons and 6 apparently positive associations, the authors stated, “Some of all of the positive findings may have arisen because of multiple testing”.

Williams A, Jalaludin B. Cancer incidence and mortality around a hazardous waste depot. Australian and New Zealand Journal of Public Health 1998;22:342-346.

In this study, cancer incidence between 1972 and 1991 and cancer mortality between 1975 and 1992 were analyzed in a population residing close to the Castlereagh Regional Waste Disposal Depot, a dumpsite in Sydney, Australia, which went into operation in 1974. Cancer incidence and mortality rates were compared with New South Wales population rates. Analysis was based on 120 diagnosed cancer cases. The study was undertaken in response to community concerns regarding the impact of hazardous waste disposal on the local health and environment. Exposure was based on residence in any of eight census districts within 3 KM of the dumpsite. Individual distances from dump site to residences were not incorporated into the analysis. No measurements or enumerations of dumpsite toxics were reported. Contamination of a potable water supply was not reported. Competing risk factors were not assessed.

For cancer incidence 1974-1991, there were 30 comparisons (14 tumor types among males and females, all cancers among males and females). Sixty additional comparisons were done using specific census year groupings. Ninety comparisons were carried out in the incidence study with no statistical adjustment for multiple comparisons.

*Results*

During the 18-year period of observation from 1974-1991, overall cancer incidence was not significantly elevated. Among individual tumor analyses, there was a statistically significant elevation of brain cancer among males. When cancer incidences were analyzed separately by census year groupings (1972-1973, 1973-1978, 1979-1983, 1984-1988, 1989-1991), statistically significant elevations were found for male brain cancer 1989-1991, female breast cancer 1979-1983, and uterine cancer 1984-1988.

Goldberg MS, Siemiatycki J, Dewar R, Desy M, Riberdy H. Risks of developing cancer relative to living near a municipal solid waste landfill site in Montreal, Quebec, Canada. Archives of Environmental Health 1999;54:291.

This study also addressed cancer incidence in the population living near the Miron Quarry municipal solid waste landfill site in Montreal. The authors used a previously completed case-control study in which individual data on key risk factors had been obtained. Cases represented thirteen sites of cancer (n=2928 subjects). There were 417 population-based controls. Although the study appears to have been undertaken in response to the concerns of local residents, a pre-existing cancer cluster had not been recognized.

Indices of exposure to gas emanating from the waste site included residence in one of four exposure zones around the site (2 high exposure zones, 1 medium exposure zone, 1 unexposed), and actual distance from the site viewed as either a continuous or categorical variable. Methane and other gases were measured at the dumpsite and within 2 KM of the site, though the results were not reported.

Residents received potable water from a municipal supply uncontaminated by the Miron Quarry. Competing risk factors were assessed individually through interviews with subjects or surrogates. Information was gathered regarding age, family income, cumulative cigarette smoking, and total alcohol consumption. For some sites of cancer, additional potential confounding variables were analyzed including ethnicity, place of birth, consumption of vitamin A, body mass index, history of hepatitis, and an index of the “dirtiness” of jobs held.

It is not possible to ascertain from the paper how many comparisons were carried out, and there is no discussion regarding use of a statistical method to adjust for multiple comparisons. Taking into account 13 separate tumors analyzed by exposure zones, as well as by distance from the waste site in .25 KM intervals, the study would have included several hundred comparisons.

While the authors report that exposure to methane, a marker for gas emanating form the waste site, was significantly higher downwind than upwind from the site, data were insufficient to detail the profile of ambient concentrations of gas around the site, and the authors cautioned that interpretation of the study results should not be made relative to gas exposure.

## Results

In the exposure zone nearest to the site, statistically non-significant elevations were found for cancers of the pancreas, liver and prostate. In the portion of that zone most frequently downwind from the dumpsite, statistically non-significant elevations of risk for pancreatic cancer and non-Hodgkins lymphoma were found. A large number of comparisons were carried out to view risk of individual cancers as a function of distance from the site, e.g. <1 KM, <1.25 KM, <1.5 KM, <2 KM. From these comparisons there were statistically non-significant excesses of pancreatic cancer for those living within 1.25 KM of the site, liver cancer for those living within 1.5 KM of the site, kidney cancer for those living within 2 KM of the site, and non-Hodgkins lymphoma for those living within 1 KM of the site.

The strength of this study is that individual data were gathered on competing risk factors. Its weakness is that some risk elevations, albeit statistically non-significant ones, may have arisen due to multiple comparisons. In addition little information was available to establish an association between residential location and biogas exposure.

**White E, Aldrich TE. Geographic studies of pediatric cancer near hazardous waste sites. Archives of Environmental Health 1999;54:390-397.**

In this study, the authors examined incidence between 1990 and 1993 of cancer among 0-17 year-old children who lived in North Carolina counties and zip code areas containing a National Priorities List (NPL) hazardous waste site. At time of writing, North Carolina contained 22 NPL sites in 18 counties. The study included 238 cases of pediatric cancer extracted from the North Carolina CCR database, which is a population-based registry. Baseline North Carolina age-adjusted cancer incidence rates for the years 1990-1993 were calculated and used as a basis of comparison for each of the analyses.

The study did not appear to have been undertaken in response to a recognized cancer cluster.

Exposure to a dumpsite was initially based on residence in a National Priorities List dumpsite-containing county. Smaller area analyses were also carried out. In one, zip-code boundaries of an NPL site defined exposure; in another exposure was defined as falling within a 1 mile radius drawn around NPL sites.

Specific toxics were not measured at the dumpsite or in residents’ homes. The authors did not report whether potable water supplies had been contaminated. There was no evaluation of competing risk factors. No statistical adjustment was made for multiple comparisons.

## Results

Most of the county SIR values were close to the null value, hovering around 1.0. None of the cancer rates by county were statistically elevated. Six of 22 zip codes had SIRs above 1.0, of which 2 showed a statistically elevated incidence. One of the two statistically elevated zip-codes contained a population of only 865 people and three pediatric cancer cases. One-third of the 22 zip codes contained no pediatric cancer cases.

Of note, only 3 cases out of the total of 238 (1.3%) fell within 1 mile of a National Priorities List Dumpsite. With the exception of elevations in two zip codes, which the authors attributed to randomness, this was a negative study.

**Knox EG. Childhood cancers, birthplaces, incinerators and landfill sites. International Journal of epidemiology 2000;29:391-397.**

In this study, the author analyzed migration patterns among children diagnosed with cancer relative to potential toxic waste exposure sources. Cases were extracted from a file of all 22,458 cancer deaths occurring before the 16^th^ birthday in Great Britain between 1953 and 1980. In 9224 cases (41.1%) the child was known to have moved at least 0.1 km between birth and death. The author determined the distance from potential toxic contamination sites (municipal incinerator, hospital incinerator, or toxic waste landfill site) of each child’s places of birth and death. Those data allowed a determination of whether the child had moved away from or toward the potential contamination site.

The study did not appear to have been carried out in response to a cancer cluster. Exposure, in effect, was based on whether the child’s death address was farther from a site of toxic contamination than the child’s birth address. Specific toxics were not measured. No information was provided regarding contamination of potable water supplies. No analysis of competing risk factors was undertaken.

The study employs a novel methodology without a control group. If one assumes that early life exposures are more relevant to carcinogenesis than are exposures later in life, then migration patterns of children with cancer might be different from migration patterns of children without cancer. If, for example, children without cancer generally moved, for whatever social or demographic reasons, from less contaminated areas to areas more proximal to potential contamination sources, and the opposite pattern were observed among children with cancer, the result might suggest that early life exposure to a site of potential contamination were associated with higher cancer risk. Without comparison to a control group, however, the net migration of children with cancer away from incinerator sites may reflect nothing other than the typical net migration from less desirable to more desirable locations as families experience income growth over the life of the child. Migration patterns among children without cancer might have demonstrated the same general pattern.

## Results

Knox found that children with cancer who had moved during their lives did so more often in a direction away from municipal and hospital incinerators, but that there was no net difference in the direction of moves relative to toxic waste landfill sites. Based on this the author suggests that there may be an association between early life exposures to waste incinerators and childhood cancer outcomes. No such effect is suggested for toxic waste landfill sites.

Pukkala E, Ponka A. Increased incidence of cancer and asthma in houses built on a former dump area. Environmental Health Perspectives 2001;109:1121.

In this study, the authors analyzed cancer incidence among inhabitants of a group of apartment buildings built atop a former waste site in Helsinki, Finland. Cancer incidence in the exposed population was compared to that of the general population in Helsinki, as well as to that occurring in an unexposed group of apartments. Expected numbers of cancer cases were based on the general Helsinki population. Mean length of follow-up for the cohort was 13.4 years.

The study did not appear to have been undertaken in response to the finding of a cancer cluster.

Exposure was based on residence in one of 12 apartment buildings built on a site of former household and industrial waste disposal. The authors sampled soil, soil gas, and ground water around the site for a wide range of toxicants. In addition, indoor air sampling was done at some apartments in the complex and compared to unexposed apartment air samples. The authors commented that exposure from the site would have occurred via dust, ambient air, and indoor air. Soil samples contained elevated concentrations of PAHs, polychlorinated biphenyls, cyanides, and some heavy metals. Indoor air measurements were similar to those from unexposed Helsinki apartments.

Residents relied on a municipal water supply and did not drink from groundwater at the dumpsite.

No individual assessment of competing risk factors was carried out. The exposed area was generally categorized as representing a low socioeconomic level. Analyses were adjusted for sex and age but not for smoking or consumption of alcohol.

There was no statistical adjustment for multiple comparisons. Incidence of overall cancer, as well as incidence of twelve specific cancers, was compared for men and women.

## Results

Eighty-eight cases of cancer were diagnosed in the dump area cohort, compared to 73 expected. Cancer excess was almost entirely attributable to males, who showed an excess of cancer of the pancreas (SIR 5.05; 95% CI: 1.38, 12.9) and cancer of the skin (SIR 4.03; 95% CI: 1.31, 9.41). SIR for cancer increased with years lived in the dump area. The authors commented that the strongest site-specific observation for cancer was the 5-fold risk of pancreatic cancer in males, but that the concentration of cases among men living a short period in one flat suggested the diseases stemmed from lifestyle habits rather than from the living environment. The authors reported that those living in the rented houses came predominantly from lower social classes with a high unemployment rate.

Jarup L, Briggs D, de Hoogh C, Morris S, Hurt C, Lewin A, Maitland I, Richardson S, Wakefield J, Elliott P. Cancer risks in populations living near landfill sites in Great Britain. British Journal of Cancer 2002;86:1732-1736.

In this study, cancer incidence among people living within 2 km of 9565 landfill sites in the UK operational between 1982 and 1997 was compared with incidence among those living more than 2 km from the sites. Data were adjusted for gender, year of diagnosis, region and socioeconomic status. The adult cancer analysis included 341,856,640 person-years, while the pediatric leukemia analysis comprised 113,631,443 person-years of observation. Available for analysis were 89,786 cases of bladder cancer; 36,802 cases of brain cancer; 21,773 cases of hepatobiliary cancer; 37,812 cases of adult leukemia; and 3973 cases of childhood leukemia. Cancers of the bladder, hepatobiliary system and brain, and leukemias were chosen due to reported associations between environmental exposures and these tumors from other studies.

Cancer incidence data were derived from registers administered by the UK Small Area Health Statistics Unit (SAHSU). The 9565 sites comprised 774 special (hazardous) sites, 7803 non-special sites and 988 sites handling “unknown” waste types. Risks for the exposed population were calculated using indirect standardization.

The study was not carried out in response to a cancer cluster. Specific toxics were not measured at the dumpsites or in residences. Contamination of potable water supplies was not addressed. Competing risk factors analyzed included age, gender, year of diagnosis, region and socioeconomic level. Aggregate data appear to have been used to assess socioeconomic level. Only five comparisons were carried out. Ninety-nine percent confidence intervals were calculated.

## Results

Adjusted for age, gender, year of diagnosis, region and socioeconomic status the rate ratio associated with bladder cancer was 1.01, brain cancer 0.99, hepatobiliary cancer 1.00, adult leukemia 0.99, and childhood leukemia 0.96. None was statistically significant. When only proximity to a special (hazardous) waste site was analyzed, no rate ratio exceeded 1.00. The authors concluded that their results did not support suggestions of excess risks of cancer associated with landfill sites.

Despite very large numbers of cases for analysis, this study found no elevation of cancer risk associated with landfill or hazardous landfill proximity. Faults of the study include that exposure was imputed based solely on 2 km proximity to a waste site at any time prior to diagnosis between 1982 and 1997, and landfills data may have been subject to errors in location, operating dates or classification of waste types. Like other studies of this type, heterogeneity of exposure within the 2 km radial regions surrounding waste sites could not be accounted for. The primary strengths of the study were its statistical power to detect an effect had one been present, and its encompassing of a large geographical region.
